# Characterization of Retinal Development in 13-Lined Ground Squirrels

**DOI:** 10.1167/tvst.11.11.17

**Published:** 2022-11-21

**Authors:** Sangeetha Kandoi, Cassandra Martinez, Dana K. Merriman, Deepak A. Lamba

**Affiliations:** 1 Department of Ophthalmology, UCSF Medical Center, University of California San Francisco, San Francisco, CA, USA; 2 Eli and Edythe Broad Center of Regeneration Medicine and Stem Cell Research, University of California San Francisco, San Francisco, CA, USA; 3 Department of Biology, University of Wisconsin Oshkosh, Oshkosh, WI, USA

**Keywords:** 13-lined ground squirrel, cone dominant, macula, retinal differentiation, retinal development

## Abstract

**Purpose:**

The cone-dominant, 13-lined ground squirrel (13-LGS) retina mimics the human central retina, but a thorough examination of retinal development in this species has not been reported. Here, the embryonic and postnatal development of the 13-LGS retina was studied to further characterize 13-LGS as a practical alternative animal model for investigating cone-based vision in health and disease.

**Methods:**

The spatiotemporal expression of key progenitor and cell type markers was examined in retinas from defined embryonic and postnatal stages using immunohistochemistry. Postnatal gene expression changes were validated by quantitative PCR.

**Results:**

The 13-LGS neuroblastic layer expressed key progenitor markers (Sox2, Vsx2, Pax6, and Lhx2) at E18. Sequential cell fate determination evidenced by the first appearance of cell-type-specific marker labeling was at embryonic stage 18 (E18) with ganglion cells (Brn-3A, HuC/D) and microglia (Iba1); at E22.5 with photoreceptor progenitors (Otx2, recoverin) followed shortly by horizontal and amacrine cells (Lhx1, Oc1) at E24 to E25.5; and at postnatal stage 15 (P15) with bipolar cells (Vsx1, CaBP5) and Müller glia cells (GS, Rlbp1). Photoreceptor maturation indicated by opsin-positive outer segments and peanut agglutinin (PNA) labeling of cone sheaths was completed at the time of eye opening (P21–P24).

**Conclusions:**

The timeline and order of retinal cell development in the 13-LGS generally matches that recorded from other mammalian models but with a stark variation in the proportion of various cell types due to cone-dense photoreceptors.

**Translational Relevance:**

This thorough examination of an emerging translationally relevant cone-dominant specie provides a baseline for future disease modeling and stem cell approach studies of human vision.

## Introduction

Inherited retinal degenerations, including age-related macular degeneration, are collectively the third leading cause of blindness in elderly humans after glaucoma and cataract.^1^ Modeling human central vision with animals is of paramount importance for investigating macular development, health, and pathology.^2^ Retinas of traditionally used nocturnal inbred rats and mice contain approximately 3% cones that are not organized into any cone-rich central region that approximates the human fovea.^3^ The retinas of certain non-human primates, such as macaques, do possess a macula, with bona fide cone-rich foveas, but non-human primates are costly and require long breeding timelines. Recently, three-dimensional retinal organoids derived from human induced pluripotent stem cells (iPSCs) have been successfully generated and appear to recapitulate the in vivo developmental timeline of retinogenesis.^4^^,^^5^ However, these human iPSC organoids fail to organize a cone-rich foveoid region. Thus, although extremely useful in their own right, these existing models for the study of progressive cone loss leave room for emerging animal models that both recapitulate human visual ecology and bear a photoreceptor mosaic similar to cone-rich regions.

One such emerging model is the ground squirrel, a diverse group of small, visually guided, diurnal rodents whose eyes possess a dichromatic 85% cone photoreceptor mosaic with a sizable cone-rich central region (the “visual streak”) resembling a large macula.^6^^,^^7^ The cone-to-rod ratio within the visual streak of the 13-lined ground squirrel (13-LGS), *Ictidomys tridecemlineatus*, is reported to be 50:1,^8^ similar to what is seen in the human central retina. The low lens-to-globe volume ratio of the 13-LGS both approximates human eye structure and also enables high-quality in vivo imaging using confocal and non-confocal split-detection adaptive optics scanning ophthalmoscopy.^9^ Hence, it is now possible to conduct non-invasive, longitudinal studies of the living 13-LGS retina in health and disease over the 6 to 7 years of the 13-LGS lifespan. However, the acuity measures from the central streak and peripheral retina have not been reported and have yet to be explored.

About 15 years ago, funding from the National Institutes of Health facilitated the establishment of a captive colony of 13-LGS at the University of Wisconsin Oshkosh (UWO). Although the 13-LGS has not been the only squirrel species used to study cone structure and function over the years, its genome has been sequenced, providing valuable molecular tools.^10^ Meanwhile, the Wisconsin colony has defined straightforward protocols for captive 13-LGS breeding and maintenance,^11^^,^^12^ thereby removing many obstacles to the use of this wild species in biomedical research. The ready availability of timed-pregnant females and elderly animals enables studies at any stage of this species’ lifespan. All of these features justify further efforts to fully characterize the squirrel model and employ it more effectively for modeling cone pathophysiology.^6^

To date, there has been no molecular and cellular description of 13-LGS retinal development. Accordingly, in this study we have used immunohistochemistry (IHC) and quantitative real-time PCR (qPCR) to set the stage for understanding key developmental events from embryonic (E) and postnatal (P) stages through eye opening, compared to adult squirrels. The data we present here fill gaps in our understanding of this emerging rodent model of central human vision, providing validated markers for future developmental and disease modeling studies.

## Methods

### Animals

All animal procedures adhered to the ARVO Statement for the Use of Animals in Ophthalmic and Vision Research and were pre-approved by the UWO Institutional Animal Care and Use Committee. The 13-LGS were bred in-house at the UWO colony. Adults were maintained on a changing light cycle matching that of Oshkosh, WI, and were overwintered in a 4°C hibernaculum prior to breeding. Pairings of males and females occurred in April as occurs in the wild, under observation; a single observed copulation event lasting 6 to 20 minutes set the timing of gestation for the embryonic samples. Newborn litters with umbilical cords still present were typically discovered on the 26th morning following copulation and set the timing for postnatal staging. All animals were euthanized under room lights by decapitation during the light phase of their photoperiod, in their euthermic active season (April–July). Eyes were collected for analysis at different embryonic stages, with E18 being the initial time point, at selected postnatal stages through eye opening, which typically occurs from P21 to P24, and from 1-year-old adult squirrels.

### Retinal Collection and Preparation

For IHC analysis, whole globes dissected from embryonic 13-LGS were immersed in freshly prepared cold 4% paraformaldehyde (Electron Microscopy Sciences, Hatfield, PA) in 0.1-M sodium phosphate buffer. The next day, fixed globes were rinsed in large volumes of buffer and stored at 4°C. Postnatal and adult eyes were similarly fixed and rinsed, except that enucleated globes were slit at the limbus before immersion into cold fixative. Rinsed globes were then shipped in 0.1-M sodium phosphate buffer on a cold pack to the University of California San Francisco (UCSF). On arrival, lens, cornea, and vitreous humor were dissected away, and the resulting eyecups were re-immersed in cold buffered 4% paraformaldehyde for 20 minutes to ensure penetration into retinal tissue for proper fixation. After rinsing with 1× phosphate-buffered saline (PBS), eyecups were passed through an ascending series of aqueous 15% and 30% sucrose for 1 hour each, by which time all eyecups had sunk to the bottom of the container vessel. Eyecups were then embedded in Tissue-Tek O.C.T Compound (Sakura Finetek USA, Torrance, CA). Cryostat sections, 10 µm thick, were collected onto clean slides and stored dry at −80°C until immunolabeling.

### Immunohistochemistry

Sections on slides were thawed at room temperature, and all subsequent steps occurred at room temperature unless otherwise noted. After rehydration with PBS, sections were permeabilized (15 minutes in PBS + 0.1% Triton X-100 + 10% normal donkey serum [NDS]) and then saturated with 10% NDS in PBS (1 hour). Sections were then incubated overnight at 4°C with primary antibodies diluted in 10% NDS in PBS (see Supplementary Table S1 for antibody details). Next day, the primary antibody solution was washed away (3 × 5 minutes in PBS), after which sections were incubated for 1 hour in Alexa Fluor–conjugated secondary antibodies made in 10% NDS in PBS (see Supplementary Table S2 for antibody details). The secondary antibody solution was removed, after which 1 µmg/mL 4′,6-diamidino-2-phenylindole (DAPI; Roche, Indianapolis, IN) in PBS was applied for 10 minutes to counterstain cell nuclei. After washing away the DAPI solution (3 × 5 minutes in PBS), slides were coverslipped using Fluoromount-G (Electron Microscopy Sciences). For Lhx1 staining, an antigen retrieval step for sections was carried out prior to blocking and staining using pH 8.0 sodium citrate buffer heated in a microwave oven for 3 to 5 minutes at 100°C. All immunoreactive cells were visualized from the central cone-rich retina on the ventral aspect closer to the optic nerve head with a ZEISS LSM 700 inverted confocal microscope (Carl Zeiss, Inc., Thornwood, NY); representative images were captured using the ZEISS ZEN microscopy software and edited using ImageJ (National Institutes of Health, Bethesda, MD). The specificity of each antibody was tested by incorporating appropriate negative controls without primary antibodies.

### Cell Type Composition

Confocal images of immunostained Brn-3A, Pax6, Vsx2, Sox2, Oc1, and Otx2 sections were montaged with the corresponding DAPI images. All DAPI-stained nuclei within three different frames per image were manually counted in an identified nuclear layer—outer nuclear layer (ONL), inner nuclear layer (INL), and ganglion cell layer (GCL)—giving total cells per layer. Then, only the cell type marker–labeled nuclei, based on the positional features within the same three frames (as with DAPI), were manually counted. This provided estimates of the proportions of different cell types, including ganglion, amacrine, bipolar, Müller glia, horizontal, and photoreceptor cells, within each retinal layer and within the neuroretina as a whole.

### Quantitative Real-Time PCR

The qPCR analysis was performed only on retinas from postnatal stage animals. Freshly enucleated globes were dissected, and the retinas were rapidly stripped out and dropped into a small volume (100–200 µL) of cold TRIzol (Zymo Research, Irvine, CA). The isolated retinas were vortexed for 1 to 2 minutes, stored frozen at −80°C, and then shipped on dry ice to UCSF. Total RNA was extracted using TRIzol per the manufacturer's instructions. Digestion of genomic DNA was carried out using TURBO DNA-*free* DNase Treatment and Removal Reagents (Bio-Rad, Hercules, CA) per the manufacturer's instruction. Total purity and concentration were determined using NanoDrop One (Thermo Fisher Scientific, Waltham, MA) for absorbance measurements at 260 and 280 nm. Total RNA (1 µg) of each postnatal stage was used for complementary DNA synthesis using a reverse transcription kit (iScript; Bio-Rad). Real-time PCR amplification was performed using iTaq Universal SYBR Green Supermix (BioRad), and the expression of each sample was detected in triplicate reactions. Thermocycler conditions were initial denaturation at 95°C for 30 seconds; 40 cycles of denaturation at 95°C for 5 seconds plus annealing at 60°C for 25 seconds; and, finally, denaturation at 95°C by 0.5°C in a real-time PCR apparatus (BioRad). The genes of interest in this study are listed in Supplementary Table S3. The primer sets were designed using PrimerQuest Tool software (Integrated DNA Technologies, Coralville, IA). Beta-actin was used as the reference gene.

### Hematoxylin and Eosin Staining

Slides containing cryosections were washed in 1× PBS to remove the Tissue-Tek O.C.T. They were then stained in Epredia Hematoxylin Gill 3 (Thermo Fisher Scientific) for 2 minutes, rinsed two times (10 dips each time) in distilled water, washed in Scott's Tap Water Substitute (2% magnesium sulfate) for 2 minutes, and rinsed again two times (10 dips each time) in distilled water. Slides were counterstained with buffered Epredia Eosin (Thermo Fisher Scientific) for 1 minute, rinsed in distilled water until the water was clear, and dehydrated in absolute ethanol slowly by dipping five times, followed by dipping five times in a 1:1 mixture of ethanol:xylene. Slides were then cleared in xylene two times for 3 minutes and immediately mounted with DPX Mountant (Electron Microscopy Sciences) and sealed with a coverslip. Hematoxylin and eosin (H&E)-stained cryosections were imaged on a ZEISS AxioPhot microscope with a SPOT Insight digital camera (Diagnostic Instruments, Sterling Heights, MI) using 2.5× and 20× objectives.

### Statistical Analysis

All statistical analyses were performed using Prism 9 (GraphPad, San Diego, CA), and the data are displayed as mean ± SEM. The comparisons among the various developmental postnatal ages were carried out using one-way or two-way analyses of variance.

## Results

### Multipotent Progenitor Cells in the Developing 13-LGS Retina

**Figure 1.**
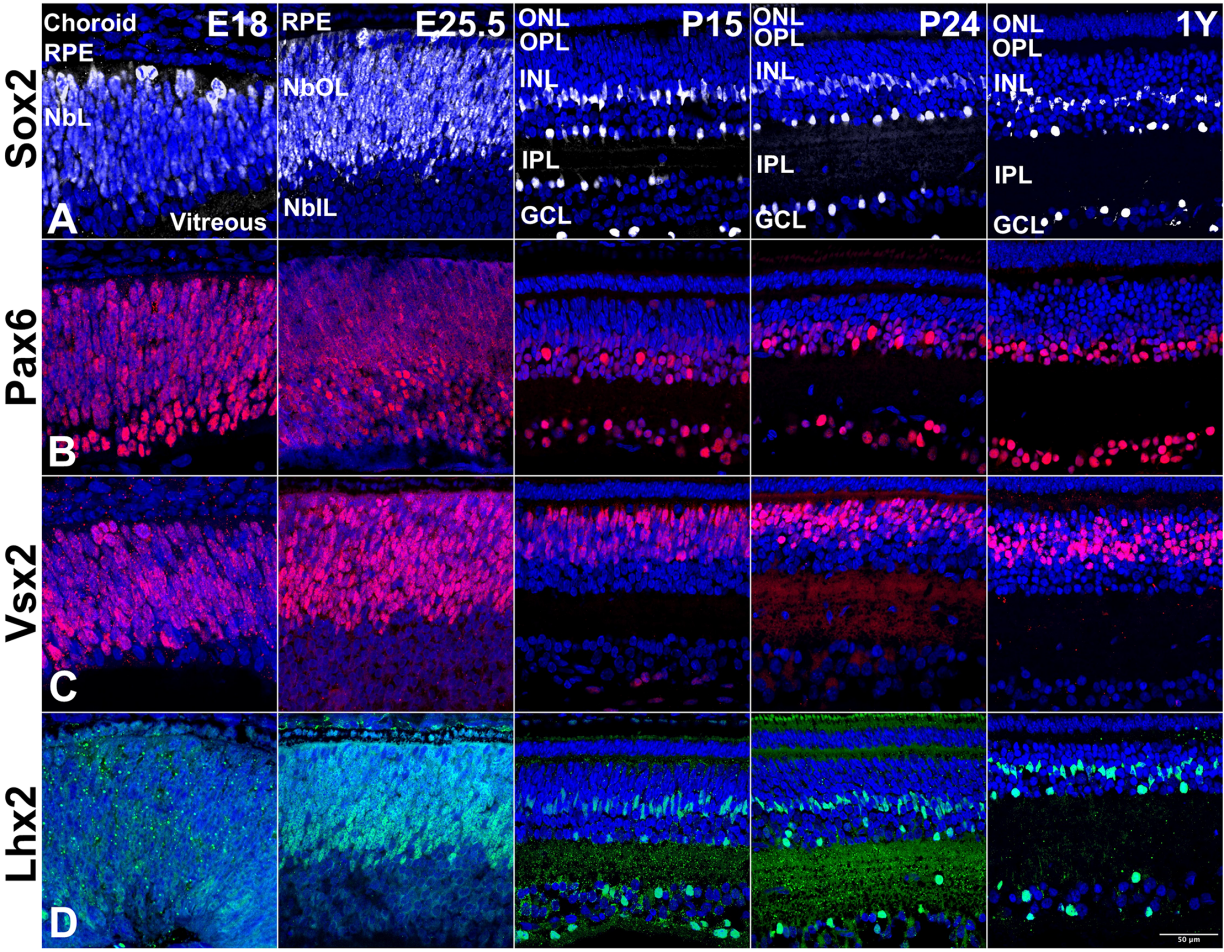
Representative 13-LGS retinal images showing progenitor marker expression. Images were collected at the indicated embryonic and postnatal days and at 1 year (adulthood). (A) Sox2+ cells (*white*) were found in the embryonic NbIL but with different levels of expression. Postnatally, they occupied a middle sublayer of the nascent INL and the inner margin of the INL, and a few were displaced in the GCL. In adult retina, they occupied locations consistent with Müller cells, amacrine cells, and ganglion cells. (B) Pax6+ cells (*red*) were distributed throughout the NbL at embryonic stages and were restricted to bipolar cells and the GCL in the postnatal and adult stages. (C) Vsx2+ cells (*red*) were located much like Sox2+ cells except that the postnatal and adult distributions were restricted to locations consistent with bipolar cells. (D) Lhx2+ cells were found throughout the NbOL at E25.5 but by 1 year were restricted to locations consistent with Müller cells and amacrine cells. DAPI counterstain marks nuclei (*blue*). E25.5, just before birth. P24, eyes open. *Scale bar*: 50 µm. RPE, retinal pigment epithelium; NbL, neuroblastic layer; NbOL, neuroblastic outer layer; NbIL, neuroblastic inner layer; ONL, outer nuclear layer; INL, inner nuclear layer; IPL, inner plexiform layer; GCL, ganglion cell layer.

First, we analyzed the presence and distribution of retinal progenitors across development using four commonly used markers known to be expressed in retinal progenitors: Sox2,^13^ Pax6,^14^ Vsx2,^15^ and Lhx2^16^ (Fig. 1). The vast majority (∼90%) of cells at E18 expressed all four progenitor markers. The expression of these markers (except Pax6) become increasingly restricted to the neuroblastic outer layer (NbOL) as embryonic retinogenesis progressed. In ≥P15 and adult 13-LGS retina, Sox2 immunoreactivity persisted in the Müller glia cells and in a subset of amacrine cells in the differentiated retinas (Fig. 1A; Supplementary Figs. S1A, S1A’). Similarly, Pax6 labeling became restricted to the cells of the GCL and a few cells of the INL, presumably amacrine cells (Fig. 1B; Supplementary Figs. S1B, S1B’). Vsx2, a homeodomain protein, was predominantly expressed within the bipolar cells of the postnatal and adult 13-LGS retina (Fig. 1C, Supplementary Fig. S1C’). Lhx2 was expressed in most neuroblastic layers (NbLs) at E18, but from P15 onward reduced Lhx2 expression was still found only in the Müller glia cells and a subset of amacrine cells (Fig. 1D, Supplementary Fig. S1D).

### Ganglion Cell Layer

**Figure 2.**
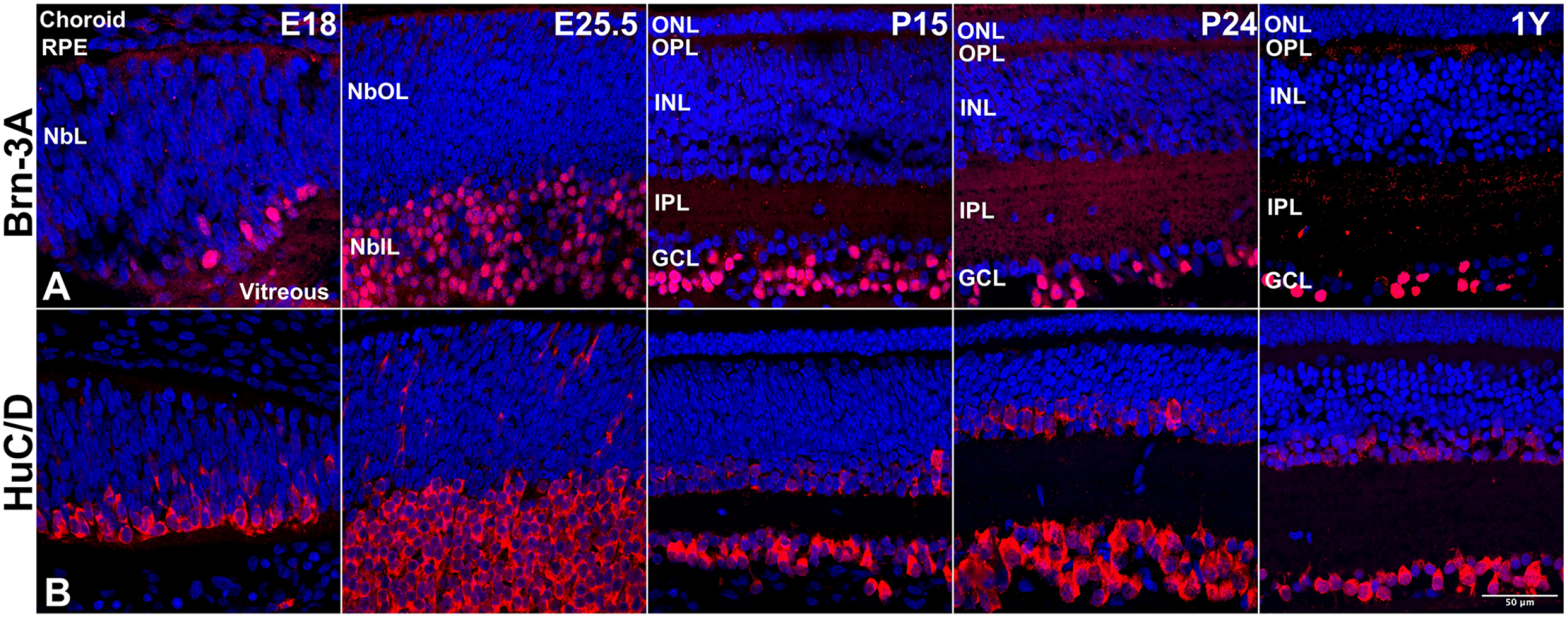
Representative 13-LGS retinal images showing retinal ganglion cell marker expression. (A) Brn-3A+ nuclei (*red*) stained cells were found in the NbIL of embryonic stages but, by adulthood, were restricted to GCs. (B) HuC/D+ cytoplasmic stained cells (*red*) were found primarily in the NbIL of embryonic stages but, by adulthood, were restricted to amacrine cells (faintly labeled) and ganglion cells. DAPI counterstain marks nuclei (*blue*). *Scale bar*: 50 µm.

The appearance of ganglion cells is the first post-mitotic event in the retina in almost all species, and 13-LGS is no exception. Brn3, a member of the Pou4f family of transcription factors, is crucial for the determination of retinal ganglion cell fate during development.^17^ In 13-LGS, Brn-3A^18^ expression was first evident at E18 and persisted through adulthood (Fig. 2A). Immunoreactivity of HuC/D^19^ was detected at E18 and E25.5 in the putative regions of the GCL overlapping with the ganglion cells. Upon further maturation, from P15 to adult, the HuC/D immunoreactivity becomes stronger and evident in both ganglion cells and in amacrine cells of the INL (Fig. 2B; Supplementary Figs. S1A, S1B’). Immunoreactive Islet-1^20^–positive cells were detected at E18 near the vitreal surface of the retina and were also faintly labeling at the apical surface of the embryonic retina at E25.5, just before birth (Supplementary Fig. S2A). The differentiation wave was seen initiating from the central to peripheral retina (Supplementary Fig. S2B) in 13-LGS similar to published reports on other species.^21^ From P10 onward through adult retina, Islet-1 expression was restricted to the ganglion, bipolar, and amacrine cells (Supplementary Fig. S2A).

### Outer Nuclear Layer

**Figure 3.**
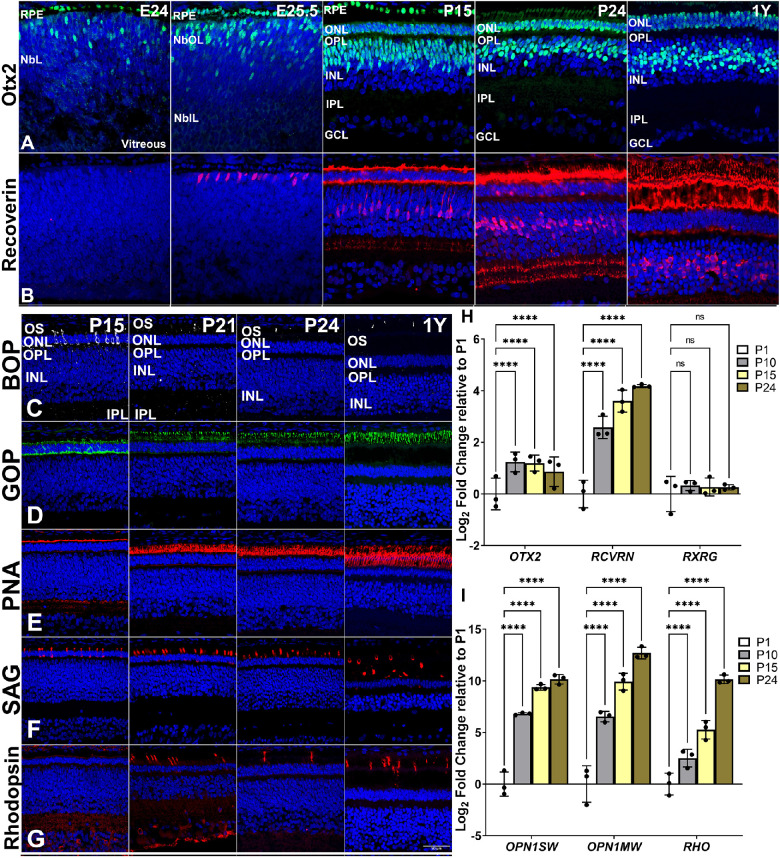
Representative 13-LGS retinal images showing photoreceptor marker expression along with postnatal gene expression data. (A) Otx2+ cells (*green*) first appeared in the NbOL at E24, 2 days before birth (and separately included the RPE). By P15, they occupied the RPE layer, the ONL, and the inner INL. In adult, Otx2+ cells were comprised of the RPE, photoreceptor cells, and a subset of bipolar cells. (B) Recoverin+ cells (*red*) first appeared in the NbOL after Otx2+ cells at E25.5, the day before birth. By adulthood, Recoverin+ cells were restricted to photoreceptor cells and a subset of bipolar cells. (C) BOP+ cells (*white*) first appeared at P15 in the ONL nuclei and in locations consistent with IS and nascent OS by P21 (onset of eye opening); label was restricted to OS, just as in the adult. (D) GOP+ cells (*green*) appeared much as did BOP+ cells but in far greater numbers, reflecting the abundance of green cones in adult 13-LGS retina. (E) PNA lectin-labeled OS sheaths (*red*) were evident at P15, corresponding to labeling of cone opsins, and achieved an adult-like size and distribution by eye opening (P21–P24). (F) SAG+ cells (*red*) were evident at P15 in locations consistent with nascent OS. (G) Rhodopsin+ cells (*red*) appeared similar to SAG+ cells. (H) Postnatal expression of early photoreceptor gene (*OTX2*, *RCVRN*, and *RXRG*) mRNA transcripts from P10 to P21, relative to P1 (*n* = 3 animals). (I) Postnatal expression of matured photoreceptor gene (*OPN1SW*, *OPN1MW*, and *RHO*) mRNA from P10 to P24, relative to P1 (*n* = 3 animals). As eye opening approached, transcription of both cone opsins and of rhodopsin increased profoundly. ****P < 0.0001. DAPI counterstain marks nuclei (*blue*). *Scale bars*: 50 µm.

Photoreceptors labeled by Otx2^22^ appeared first at E21, indicating the earliest time point of photoreceptor genesis in the central retina (Supplementary Fig. S2B), with widespread expression by E24 (Fig. 3A). Otx2 was also expressed in the retinal pigment epithelium (RPE) cell layer (noticed first at E18, data not shown) and persisted in bipolar cells in postnatal 13-LGS retinas. Recoverin,^23^ which marks maturing rods and cones, was first observed at E25.5 just before birth, representing the turning on of phototransduction machinery and morphogenesis (Fig. 3B). Intense immunoreactivity of recoverin-positive cells was apparent in photoreceptors along with moderately labeled bipolar-resembling cells of the INL from P15 to adulthood. Visual pigment proteins for S-cones (BOP), M-cones (GOP), and rods (rhodopsin)^24^ were labeled in the outer retina by P15 (Figs. 3C, 3D, 3G), but mature restriction of labeling to outer segments (OSs) took a few more days to achieve (i.e., by P21), the typical onset of eye opening. This change occurred approximately 3 weeks after the transcripts were detected by qPCR (Fig. 3I). Immunolabeling for S-arrestin,^25^ a key rod phototransduction protein (S-antigen visual arrestin [SAG]) (Fig. 3F), was detected in the OSs of maturing rod photoreceptors at P15 and was maintained through adulthood. Cone-specific peanut agglutinin (PNA) lectin^26^ labeling of the interphotoreceptor matrix around inner segments (ISs) and OSs was expressed at P21 (Fig. 3E). Overall, and as expected, the 13-LGS retina was highly enriched with M-cones compared to S-cones and rods.^8^^,^^9^ We confirmed this previous reported distribution of cones and rods on flat mounts of P24 retinal tissue from central and ventral mid-peripheral retina which has a higher concentration of rods^8^ (Supplementary Fig. S3).

Consistent with the IHC data, the gene expression profiles of *OTX2* and *RXRG*^27^ mRNA transcripts were detected throughout the postnatal stages, albeit at low levels, implicating the end of photoreceptor progenitor and cone development during the embryonic stages. The maturation of photoreceptors was evident by the expression of *RCVRN* transcripts showing a 32-fold increase in the postnatal retina from P1 to P24 (Fig. 3H). The onset of *OPN1SW*, *OPN1MW*, and *RHO* transcripts was detected from low to high from P1 to P24 for the two cone subtypes and the single rod type (Fig. 3I).

### Inner Nuclear Layer

**Figure 4.**
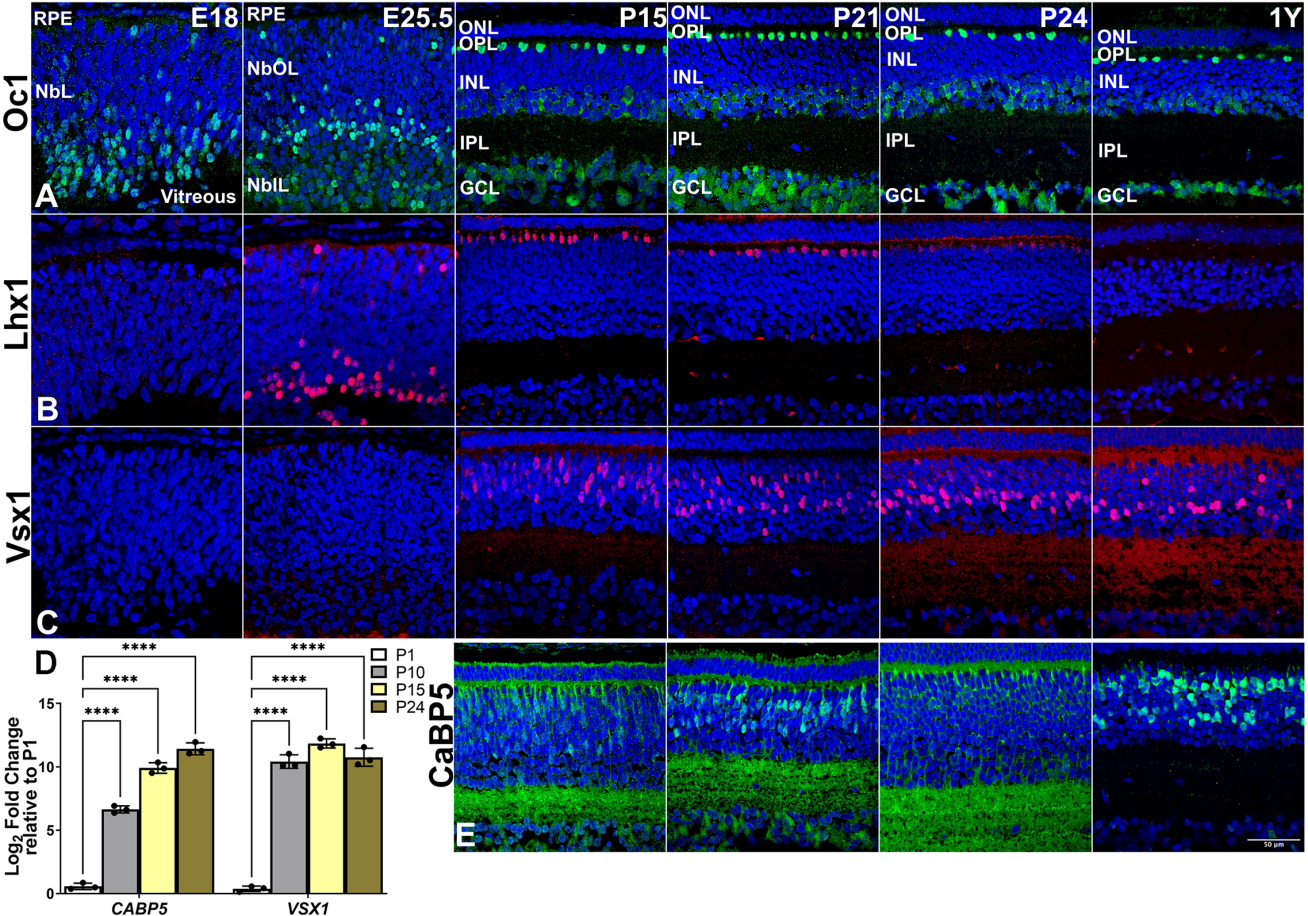
Representative 13-LGS retinal images of INL neuron marker expression along with postnatal gene expression data. (A) Oc1+ cells (*green*) appeared in the NbIL at E18. By P15, this label was restricted to locations consistent with horizontal cells, amacrine cells, and ganglion cells, much as seen in the adult. (B) Lhx1+ cells (*red*) appeared later than Oc1+ cells, on the day before birth. As eye opening approached, label persisted in locations consistent with horizontal cells and amacrine cells, but at the completion of eye opening (P24) this transient labeling had disappeared. (C) Vsx1+ cells (*red*) appeared later than Lhx1+ cells, at P15, in locations consistent with a subset of bipolar cells. This labeling pattern persisted into adulthood. (D) Postnatal expression of bipolar cell gene (*CABP5* and *VSX1*) mRNA transcripts from P10, P15 and P24 relative to P1 (*n* = 3 animals). *CABP5* mRNA is substantially elevated at P10 and continues to upregulate as eye opening approaches, whereas elevated levels of *VSX1* mRNA are stable over the time frame of P10 to P24. (E) CaBP5+ cells (*green*) appeared similarly to Vsx1+ cells but labeled a different subset of developing bipolar cells in a pattern that persisted in adulthood. ****P < 0.0001. DAPI counterstain marks nuclei (*blue*). *Scale bar*: 50 µm.

The three types of second-order neurons residing in the INL are horizontal cells, bipolar cells, and amacrine cells. The INL synapses with photoreceptor and ganglion cells in the outer plexiform layer (OPL) and inner plexiform layer (IPL), respectively, for transmission of visual signals. Onecut1 (*OC1/HNF6*) is a transcription factor driving cone, ganglion, and horizontal cell differentiation. We observed Oc1 from the first wave of neurogenesis appearing at E18. At P15, when the plexiform layers were clearly developed, Oc1 was strongly expressed in horizontal cells close to the OPL boundary and weakly expressed in ganglion cells and, interestingly, in amacrine cells (Fig. 4A). Histological analysis showed the first expression of Lhx1,^28^ a horizontal cell marker, in scattered cells at the basal surface of the embryonic retina at E25.5 (Fig. 4B). Postnatally, the committed post-mitotic Lhx1-positive horizontal cells were confined to a single layer in the INL, close to the OPL, by P15. Weak Lhx1 immunoreactivity was still detected in the horizontal cells at P24 but disappeared in the adult retina, suggesting a limited role in horizontal cell maturation. Bipolar cells expressing Vsx1^29^ protein were detected at P15 (Fig. 4C; Supplementary Figs. S1C, S1C’), whereas *VSX1* mRNA was detected at P10, peaked by P15, and further declined at P24 (Fig. 4D). Immunolabeling of CaBP5^30^ localizing post-mitotic bipolar cells in the INL was seen from P15 through adulthood (Fig. 4E, Supplementary Fig. S1C). The onset of *CABP5* gene expression, another bipolar cell marker, was seen at P10, and its relative expression peaked gradually from P1 to P24, through P10 and P15 (Fig. 4D).

### Glial Cells

**Figure 5.**
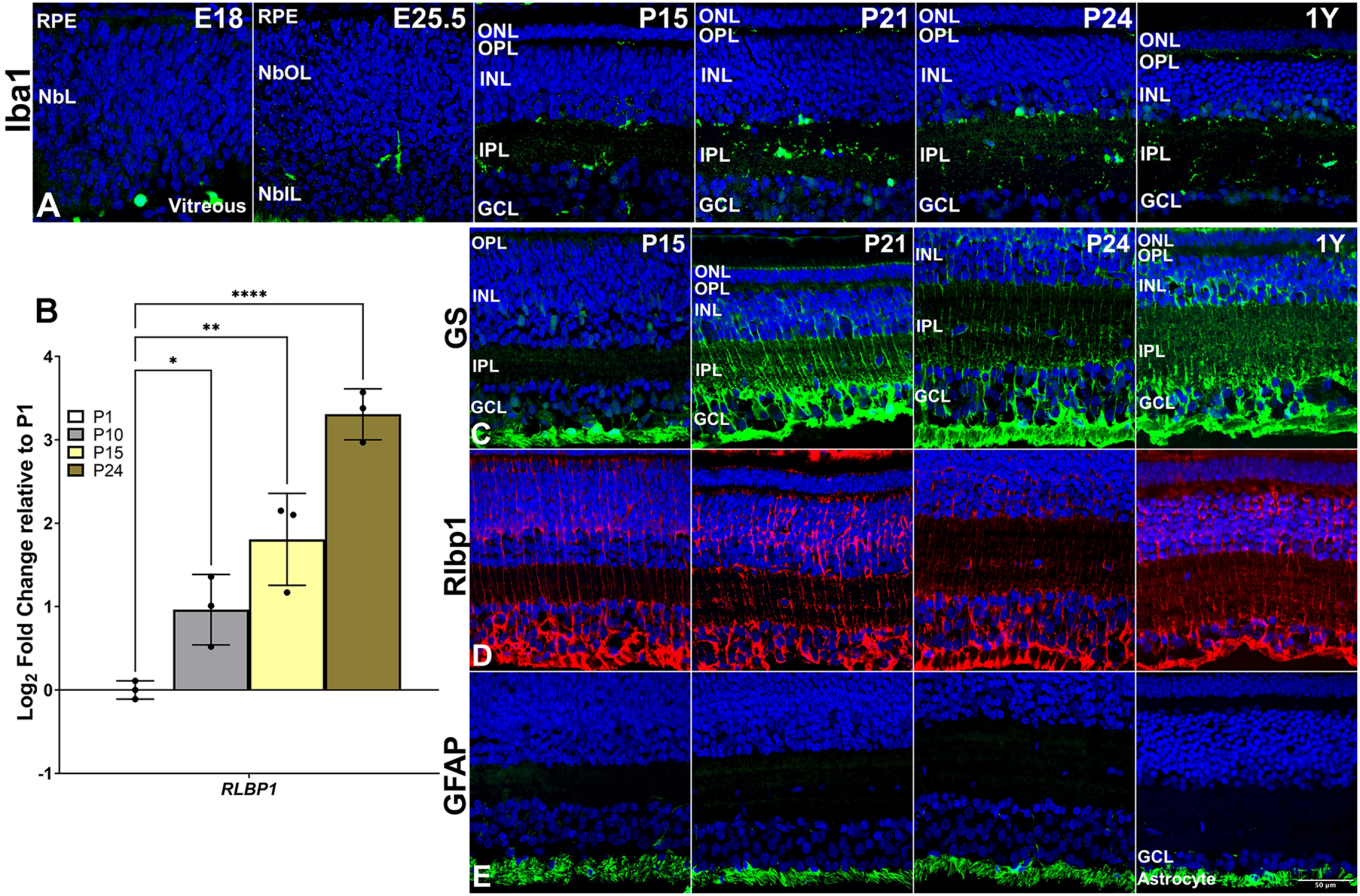
Representative 13-LGS retinal images showing microglial and macroglial marker expression along with postnatal gene expression data. (A) Iba1+ amoeboid cells (*green*) appeared in the NbIL at E18, but these labeled cells had a ramified appearance the day before birth (E25.5). At P15, label was restricted to the inner margin of the INL, the nascent IPL, and occasional profiles in the GCL. Labeled amoeboid profiles reappeared in these locations at the onset (P21) and completion (P24) of eye opening but were not observed in the retinas of healthy adults (1 year). (B) Postnatal expression of macroglial *RLBP1* gene mRNA transcript increased steadily from P10 to P24, relative to P1 (*n* = 3 animals). (C) GS+ cells (*green*) were observed at P15 but only in the astrocytes and in a few Müller cell endfeet located in the GCL. By the onset of eye opening (P21), the label extended from the inner limiting membrane to the outer margin of the ONL, in a distribution consistent with the mature form of Müller cells. (D) Rlbp1+ cells (*red*) at P15 appeared as nearly mature Müller cells extending from the ONL to the inner limiting membrane, changing little as development proceeded to the adult. (E) GFAP+ cells (*green*) at P15 and all later stages appeared solely in the GCL, consistent with the location of astrocyte and Müller cell endfeet, although this label did not recapitulate the shape of Müller cell endfeet revealed by the probes for GS and Rlbp1, leading us to suggest it is within astrocytes alone. *P < 0.05, **P < 0.01, and ****P < 0.0001. DAPI counterstain marks nuclei (*blue*). *Scale bar*: 50 µm.

Retinal glial cells are commonly classified into macroglia and microglia and promote homeostasis in the neurons. Microglia expressing allograft inflammatory factor 1/ionized calcium-binding adapter molecule 1 (AIF-1/Iba1)^31^ were identified in the retina of 13-LGS as early as E18 in the activated amoeboid or active stage (Fig. 5A). By E25.5 just before birth, the localization of Iba1-positive cells revealed a morphological change in the resting ramified form that predominated at P15 and in the adult retina. Interestingly, a mix of activated amoeboid and resting ramified microglia was observed at P21 and P24 during eye opening, suggesting a brief stress response to this event. Furthermore, the ramified form of microglia persisted in the plexiform layers with the majority of them occupying the IPL in the postnatal and adult stages. Retinaldehyde binding protein 1 (Rlbp1)^32^ and glutamine synthetase (GS)^33^ specifically and strongly labeled both types of 13-LGS macroglia: the astrocytes within the nerve fiber layer and the Müller cells that spans the retina from the inner limiting membrane to the outer limiting membrane (Figs. 5C, 5D; Supplementary Figs. S1A’, S1D). Astrocytes marked by Rlbp1 and GS were detected as early as P15 and P21, respectively (Figs. 5C, 5D). GFAP-expressing astrocytes^34^ were seen at P15 onward (Fig. 5E). The mRNA expression levels of *RLBP1* were detected as early as P1, and their expression showed a sharp ∼8-fold increase at ∼3 weeks of birth from P1 to P24 (Fig. 5B).

### Plexiform Layers

**Figure 6.**
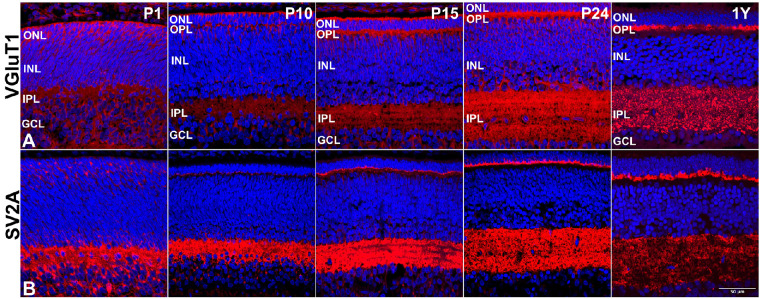
Representative 13-LGS retinal images showing synaptic marker expression. (A) VGluT1+ profiles (*red*) appeared in inner and outer retina the day after birth (P1). As the retinal layers became more organized, label in the nascent OPL was initially bilaminar but, by the completion of eye opening (P24), it attained the normal shallow single-layer characteristic of the adult of this species. Faint label was seen in the IPL as soon as it could be discerned but was robust by P24 just as in the adult. (B) SV2A+ profiles (*red*) followed a similar trajectory as did VGlut1+ profiles, except that IPL label was more robust for this marker at every stage. DAPI counterstain marks nuclei (*blue*). *Scale bar*: 50 µm.

The differentiation of the extremely ordered retinal laminae was evident with the emergence of the two plexiform layers, as detected with immunocytochemical staining for VGluT1^35^ (Fig. 6A) and SV2A^36^ (Fig. 6B). The IPL is partially developed at birth, and maturation progresses postnatally. In contrast to this, the OPL forms postnatally beginning at P10 to a mature-appearing thickness by the completed eye-opening stage of P24.

### Fraction of Cell Types in the Retina at Eye Opening

An electrophysiological study of the California ground squirrel from eye opening onward^37^ demonstrated functional maturation in that closely related squirrel species from eye opening to P70 to P80. Because the three nuclear layers of 13-LGS were readily apparent at eye opening, typically completed by all pups by P24, we estimated cell proportions within each layer and in the retina as a whole using cell-type-specific markers as follows. Brn-3A+ ganglion cells represented 38% of the GCL, Pax6+ amacrine cells were 41%, Vsx2+ bipolar cells were 45%, Sox2+ Müller glia cells were 8%, and Oc1+ horizontal cells were 2%. All ONL cells were Otx2+, representing 100% photoreceptors. From this analysis, the total fraction of each marker-specified retinal cell type within 13-LGS retina came out to 6% ganglion cells, 29% amacrine cells, 29% bipolar cells, 6% Müller glia, 1% horizontal cells, and 17% photoreceptor cells in the retina. The rest included displaced amacrine cells in the GCL and microglia that were not specifically counted.

### Retinal Morphological Observation

Finally, we carried out a morphological analysis of 13-LGS retinal tissue at embryonic, postnatal, and adult stages by H&E staining of tissues. Qualitative analysis of the retinal tissue sections by H&E revealed the neuroblastic layers in embryonic stages (Supplementary Fig. S4). In postnatal stages, we focused the analysis on the dorsal, ventral, and optic nerve head region. Although the inner plexiform layer is evident around E25.5, the outer plexiform layer was first observed in the dorsal retina at P5. Inner retinal vascular plexus in the IPL is clearly observed by P24 (eye opening) (Supplementary Fig. S4).

## Discussion

**Figure 7.**
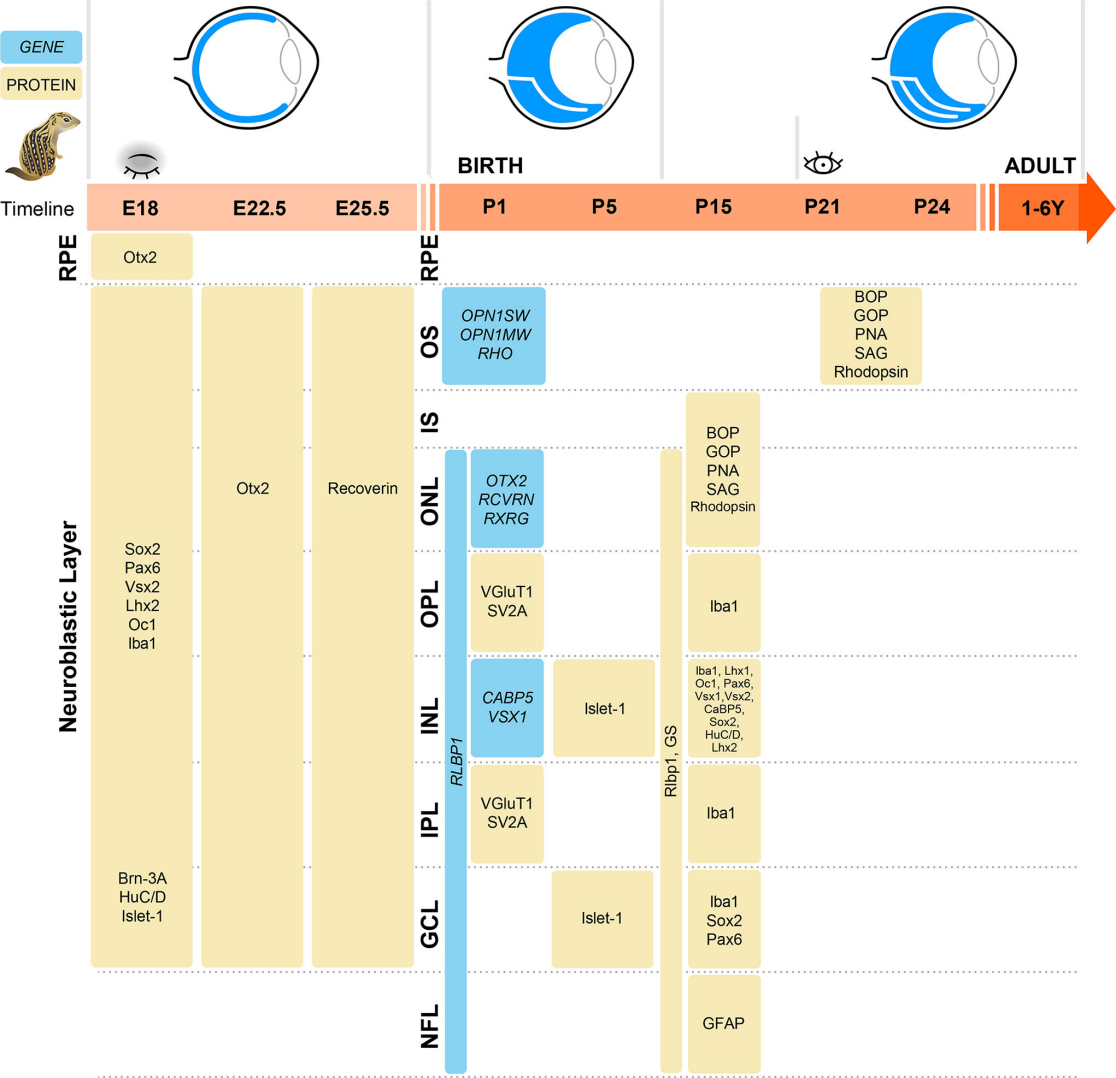
Schematic representation of retinal development cell type marker localization patterns in the cone-dominant 13-LGS starting at E18 through postnatal and adult (1 year old). BOP/*OPN1SW*, short-wave-sensitive opsin1; Brn-3A, brain-specific homeobox/POU domain protein 3A; CaBP5, calcium-binding protein 5; GFAP, glial fibrillary acidic protein; GOP/*OPN1MW*, medium-wave-sensitive opsin 1; GS, glutamine synthetase; Oc1, one cut homeobox 1; HuD, Hu-antigen D; Iba1, ionized calcium-binding adapter molecule 1; Islet-1, insulin gene enhancer protein ISL-1; Lhx1, LIM homeobox protein 1; Lhx2, LIM homeobox protein 2; *OTX2*, orthodenticle homolog 2; Pax6, paired box protein 6; PNA, peanut agglutinin; *RHO*, rhodopsin; Rlbp1, retinaldehyde-binding protein 1; *RXRG*, retinoic acid receptor RXR-gamma; SAG, S-arrestin/rod photoreceptor arrestin; SV2, synaptic vesicle glycoprotein 2A; VGluT1, vesicular glutamate transporter 1; Vsx1, visual system homeobox 1; Vsx2/Chx10, visual system homeobox 2/homeobox protein CHX10.

Previous studies have examined adult cell types in 13-LGS (and other ground squirrel) retinas using diverse methods, including electron microscopy,^38^ non-invasive imaging,^38^ northern blot hybridization,^39^ western blotting,^40^ and immunolabeling.^41^^–^^43^ Indeed, the ground squirrel visual system has been a useful model for a very long time,^6^^,^^44^ yet its retinal development literature consists of a single paper.^45^ Here, we have provided a comprehensive analysis of retinal developmental dynamics in the cone-dominant 13-LGS retina, starting at E18, and including cell-type marker localization patterns at eye opening and in the adult (Fig. 7). Additionally, the availability of the predicted genome database has allowed us to analyze postnatal gene expression in concert with the protein level using IHC. We found that mRNA for a gene detected by qPCR could significantly precede the corresponding protein detected by antibody labeling.

One of the biggest challenges in the characterization of an emerging model species is the validation of antibodies that can cross-react with that species’ proteins, and we did face some limitations in marker labeling. For example, Brn-3A labels only a subset of all ganglion cells, leaving the other subtypes undetected. Furthermore, antibodies for early rod-specific markers, neural retina leucine zipper and photoreceptor-specific nuclear receptor, did not detectably react with the 13-LGS protein. Nonetheless, we have been able to generate a validated list (Supplementary Table S1) for 13-LGS retina that can be utilized for future studies.

Our description of spatiotemporal expression patterns of retinal genes and proteins in the 13-LGS retina provides the basis for comparing developmental dynamics across species, including traditional nocturnal rodent models of vision, and is also a foundation for diverse studies that aim to use this emerging animal model of complex degenerative diseases such as age-related macular degeneration. Furthermore, given advances in iPSC technologies, our characterization of in vivo development will help to stage three-dimensional retinal organoids derived from 13-LGS iPSCs.^46^^,^^47^

We observed that, similar to other species,^48^ ganglion cells were the first neurons to differentiate in the 13-LGS retina, followed by horizontal, photoreceptor, amacrine, bipolar, and Müller glia cells. As in mouse retina, photoreceptor maturation signaled by opsin expression occurred postnatally prior to eye opening in 13-LGS, between P15 and P21. Most 13-LGS photoreceptors took on a cone fate choice, with M-cones outnumbering S-cones. Our data on adult 13-LGS cones closely match the previous ex vivo and adaptive optics scanning laser ophthalmoscopy (AOSLO) live imaging data from the species,^9^ as well as data from another cone-dominant ground squirrel, *Spermophilus beecheyi* (California ground squirrel), which has 14 M-cones for each S-cone.^8^ We observed this M-cone dominance in flatmount analyses, as well, along with a differential distribution of rods, as previously reported.^8^ It would be interesting and informative to study these distributions differentially in various retinal regions by carrying out focal qPCR or, better yet, single-cell transcriptomic analysis from different regions of the developing 13-LGS retina. Interestingly, all 13-LGS photoreceptors occupy a relatively thin ONL just two to three somata thick compared to mouse, rat, and human ONLs that are eight to 10 somata thick. This may represent the paucity of rods in 13-LGS, as a thicker ONL is a feature of rod-dominant species. A thin ONL much like that of the 13-LGS (85% cones) is also observed in the cone-dominant tree shrew (>95% cones),^49^ a species in which photoreceptors also mature by eye opening.^50^

The orchestrated process of retinogenesis is widely conserved among species, although some key differences have been identified depending on the dominant cell type present. Interestingly, in 13-LGS we did not observe any obvious difference in birth-dating of the retina compared to mouse developmental timelines, but there was a glaring difference in the relative percentage of various retinal cells, the biggest being the percentage of photoreceptors. Although the mouse retina has approximately 80% photoreceptors, most of which are rod photoreceptors,^51^ the photoreceptors in 13-LGS account for fewer than 20% of all cells. As a result, amacrine and bipolar cells account for over 29% of cells as opposed to 7% in mice retina.^51^ On the other hand, bipolar cells still make up close to 40% of the inner nuclear layer, similar to mice. Interestingly, even in mice, the cone bipolar cells vastly outnumber rods by 2.5-fold despite the relatively small fraction of cones.^52^ Based on the laminar organization of axon terminals in bipolar cells, as many as 13 different types of cone bipolar cells have been identified in adult ground squirrels.^53^^,^^54^ It will be interesting to understand the cone versus rod bipolar cell ratios and synaptic connectivity in 13-LGS.

In conclusion, to our knowledge, this is the first published study of ground squirrel in vivo retinogenesis employing whole retina transcriptome and localized proteome changes in distinct cell types. Molecular and morphological profiling of the 13-LGS retina will facilitate the robust use of these animals for studying retinal development and degeneration. Furthermore, we have identified a validated set of specific markers that can reliably identify the specific retinal cell types in future developmental and disease studies for this unique retinal resource.

## Supplementary Material

Supplement 1

Supplement 2

Supplement 3

Supplement 4

Supplement 5
