# Supplementary figures and images for: Characterization of Retinal Development in 13-Lined Ground Squirrels
Source: Transl Vis Sci Technol. 2022 Nov 21;11(11):17. doi: 10.1167/tvst.11.11.17 (PMC9695149; doi:10.1167/tvst.11.11.17)

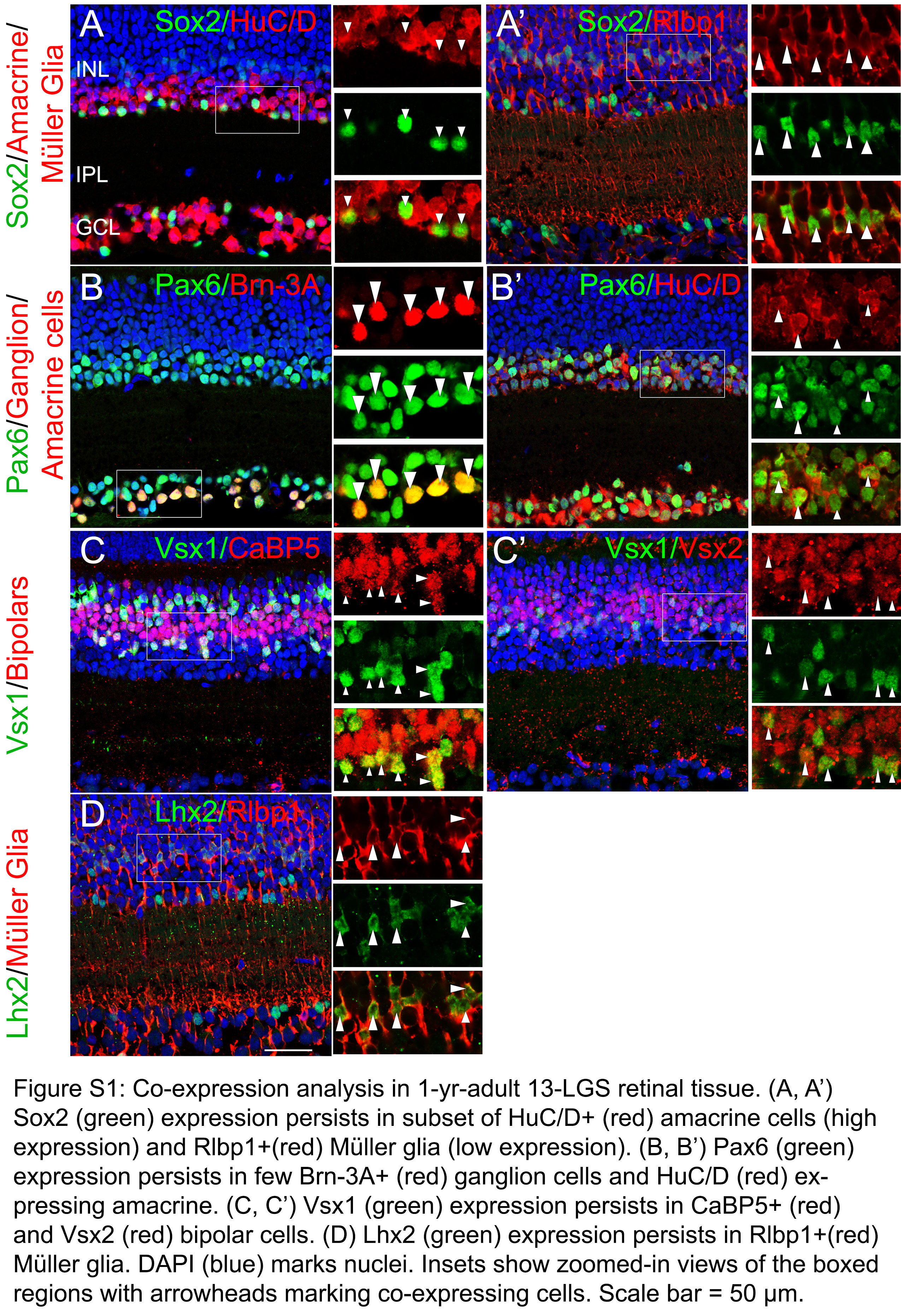

Supplement: Supplement 1 [file tvst-11-11-17_s001.jpg]

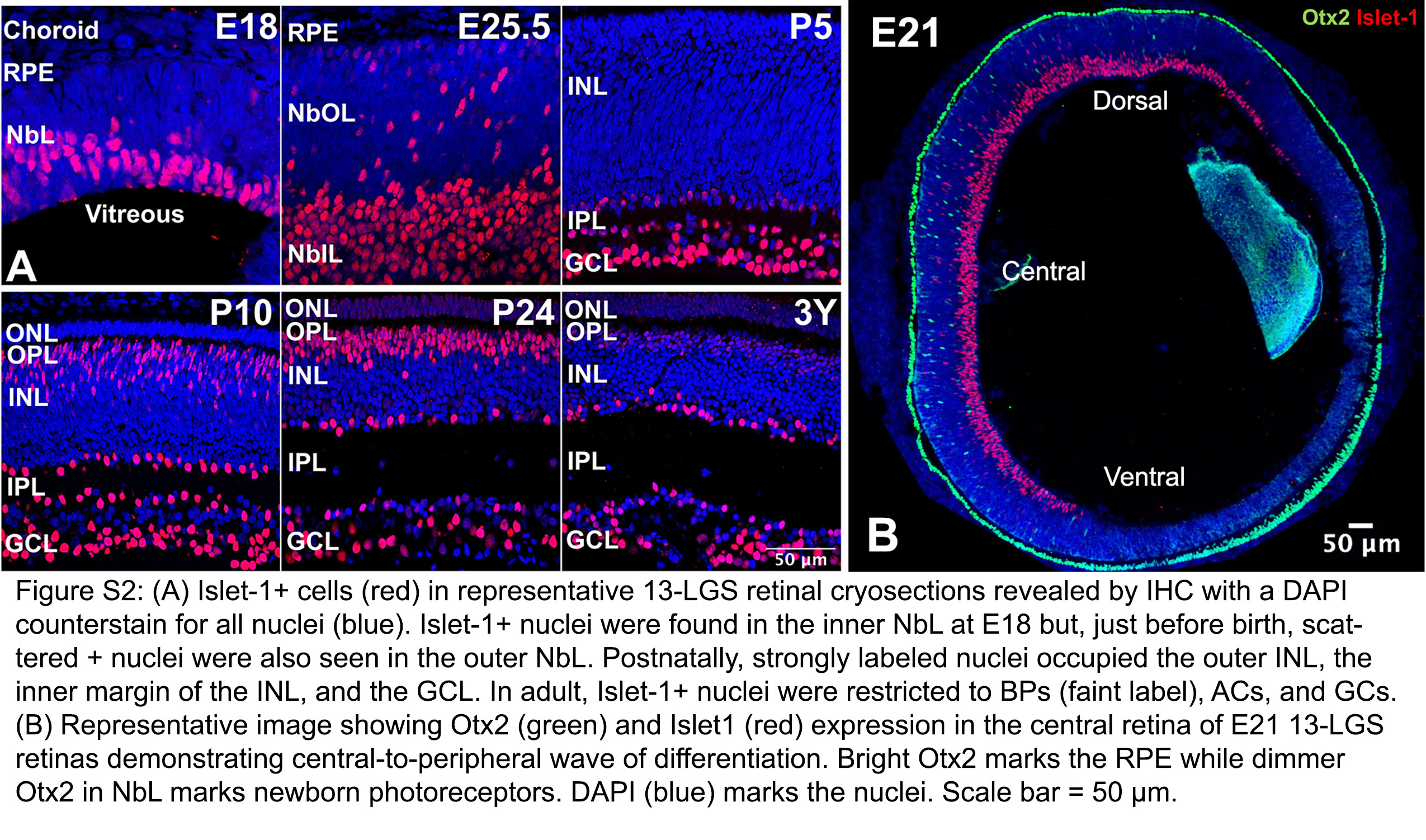

Supplement: Supplement 2 [file tvst-11-11-17_s002.jpg]

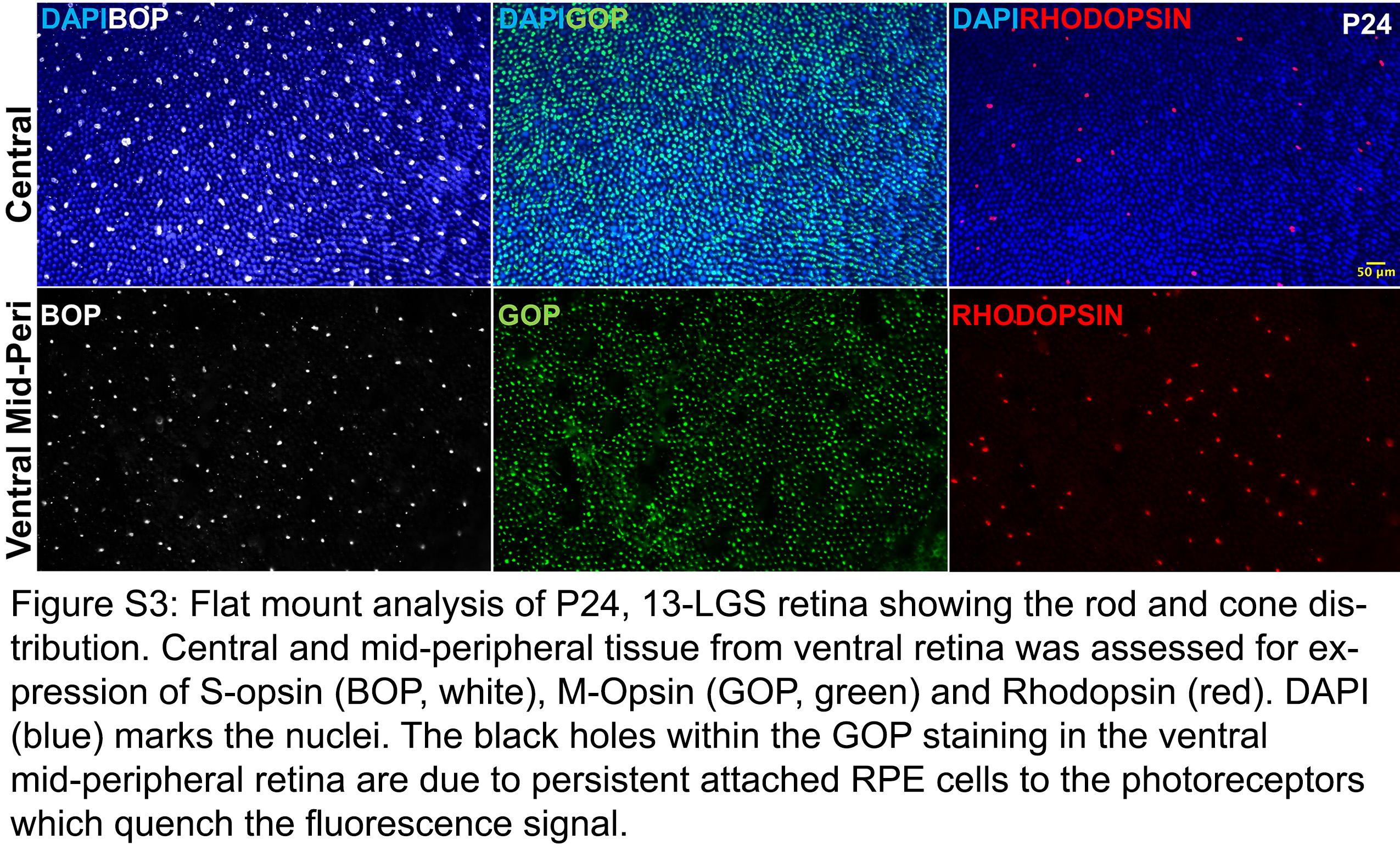

Supplement: Supplement 3 [file tvst-11-11-17_s003.jpg]

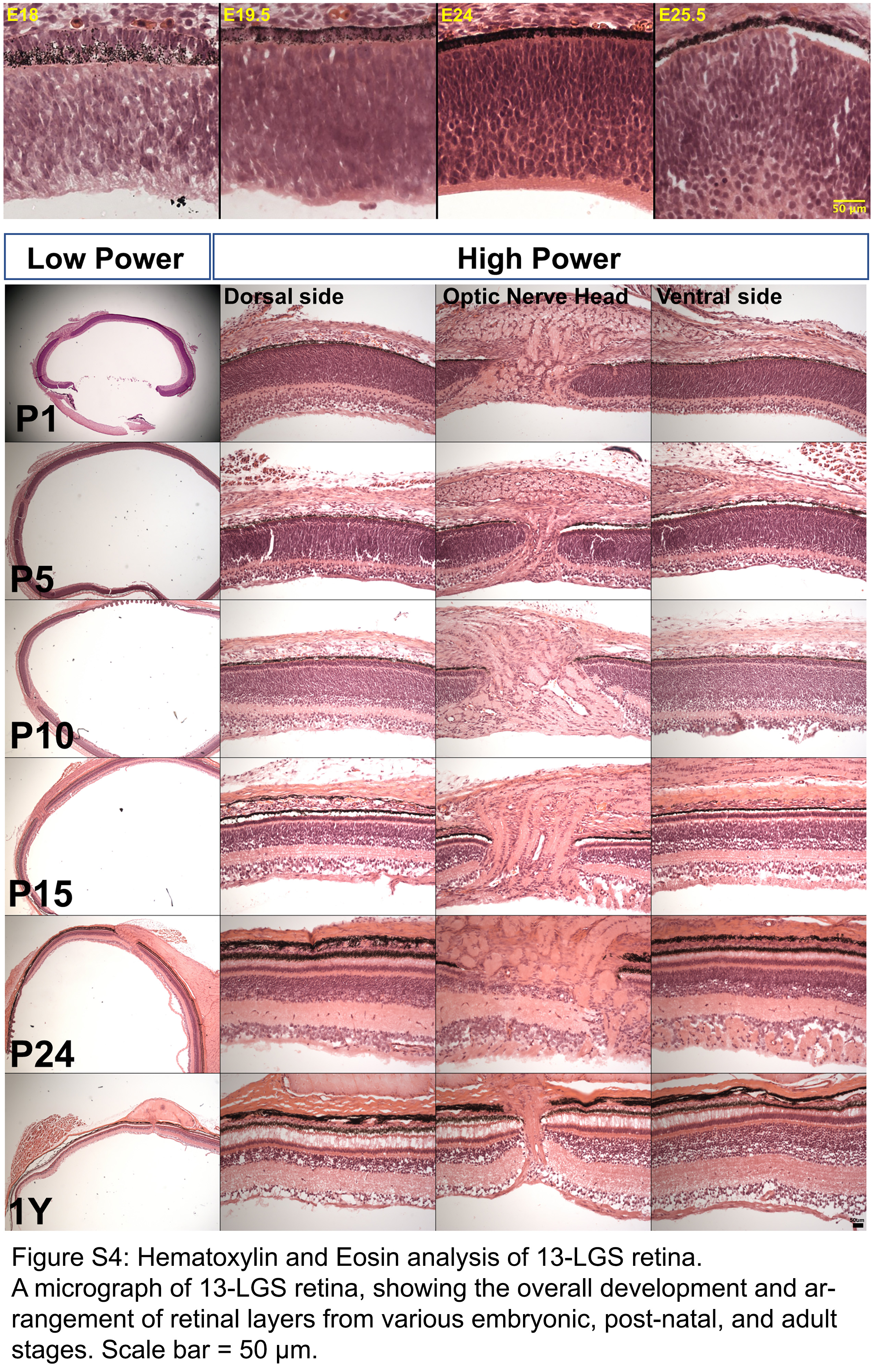

Supplement: Supplement 4 [file tvst-11-11-17_s004.jpg]
