# Supplementary material for: Characterization of Retinal Development in 13-Lined Ground Squirrels
Source: Transl Vis Sci Technol. 2022 Nov 21;11(11):17. doi: 10.1167/tvst.11.11.17 (PMC9695149; doi:10.1167/tvst.11.11.17)
Supplement: Supplement 5 [file tvst-11-11-17_s005.pdf]

Supplementary Table 1. Primary Antibody List.

Protein Name	Host	Target Cell	Dilution	Source	Cat. No.
AIF-1/Iba1	Goat	Microglia	1:100	Novus Biologicals	NB100-1028
BOP	Rabbit	Cone photoreceptors outer segments (S-subtype)	1:100	EMD Millipore	AB5407
Brn-3A	Mouse	Ganglion cells	1:100	Santa Cruz Biotechnology, Inc.	sc-8429
CaBP5	Rabbit	Bipolar cells	1:400	Gift from Palczewsky Lab	-
GFAP	Rabbit	Astrocytes	1:500	Dako	Z0334
GOP	Rabbit	Cone photoreceptors outer segments (M-subtype)	1:250	EMD Millipore	AB5405
GS	Rabbit	Astrocytes, Müller cells	1:2500	Abcam	ab49873
HNF-6/Oc1	Rabbit	Ganglion cells, Amacrine cells, Horizontal cells	1:50	Santa Cruz Biotechnology, Inc.	sc-13050
HuC/D	Mouse	Ganglion cells, Amacrine cells	1:100	Life Technologies (Invitrogen)	A-21271
Islet-1	Mouse	Ganglion cells, Amacrine cells, Bipolar cells	1:10	DSHB	39.4D5
Lhx1	Mouse	Horizontal cells	1:25	DSHB	4F2-s
Lhx2	Goat	Progenitor cells, Müller cells, Amacrine cells	1:200	Santa Cruz Biotechnology, Inc.	sc-19344
Otx2	Goat	Bipolar cells, Photoreceptors, RPE	1:400	R&D Systems	BAF1979
Pax6	Mouse	Progenitor cells, Ganglion cells, Amacrine cells	1:100	DSHB	PAX6-s
Pax6	Rabbit	Progenitor cells, Ganglion cells, Amacrine cells	1:50	BioLegend	901301
PNA	Biotin	Cone photoreceptors	1:1000	Vector Laboratories	B-1075
Recoverin	Rabbit	Photoreceptors	1:500	EMD Millipore	AB5585
Rhodopsin	Mouse	Rod photoreceptors outer segments	1:250	Santa Cruz Biotechnology, Inc.	sc-57432
Rlbp1	Mouse	Astrocytes, Müller cells	1:200	Santa Cruz Biotechnology, Inc.	sc-59487
SAG	Mouse	Rod photoreceptors outer segments	1:200	Santa Cruz Biotechnology, Inc.	sc-166383
Sox2	Goat	Progenitor cells, Müller cells, Amacrine cells	1:400	R&D Systems	AF2018

SV2A	Mouse	Plexiform layers	1:200	DSHB	SV2
VGluT1	Guinea Pig	Plexiform layers	1:250	EMD Millipore	AB5905
Vsx1	Mouse	Bipolar cells	1:200	Santa Cruz Biotechnology, Inc.	sc-393699
Vsx2 / Chx10	Mouse	Progenitor cells, Bipolar cells	1:100	Santa Cruz Biotechnology, Inc.	sc-365519

Abbreviations: AIF-1/Iba1, Allograft inflammatory factor 1/Ionized calcium-binding adapter molecule 1; BOP, short-wave-sensitive opsin1; Brn-3A, brain-specific homeobox/POU domain protein 3A; CaBP5, calcium-binding protein 5; GFAP, Glial fibrillary acidic protein; GOP, medium wave sensitive opsin 1; GS, Glutamine synthetase; HNF-6/Oc1, Hepatocyte nuclear factor 6/one cut homeobox 1; HuD, Hu-antigen D; Islet-1, Insulin gene enhancer protein ISL-1; Lhx1, LIM homeobox protein 1; Lhx2, LIM homeobox protein 2; OTX2, Orthodenticle homolog 2; Pax-6, Paired box protein Pax-6; PNA, Peanut agglutinin; Rlbp1, retinaldehyde-binding protein 1; SAG, S-arrestin/rod photoreceptor arrestin; SV2, synaptic vesicle glycoprotein 2A; VGluT1, Vesicular glutamate transporter 1; Vsx1, Visual system homeobox 1; Vsx2/Chx10, Visual system homeobox 2/Homeobox protein CHX10.

Supplementary Table 2. Secondary Antibody List.

Species	Target	Fluorochrome	Dilution	Source	Cat. No.
Donkey	Anti-mouse	Alexa Fluor 488	1:250	Invitrogen	A-21202
Donkey	Anti-mouse	Alexa Fluor 555	1:250	Invitrogen	A-31570
Donkey	Anti-mouse	Alexa Fluor 647	1:250	Invitrogen	A-31571
Donkey	Anti-rabbit	Alexa Fluor 488	1:250	Invitrogen	A-21206
Donkey	Anti-rabbit	Alexa Fluor 555	1:250	Invitrogen	A-31572
Donkey	Anti-rabbit	Alexa Fluor 647	1:250	Invitrogen	A-31573
Donkey	Anti-Goat	Alexa Fluor 488	1:250	Invitrogen	A-11055
Donkey	Anti-Goat	Alexa Fluor 555	1:250	Invitrogen	A-21432
Donkey	Anti-Goat	Alexa Fluor 647	1:250	Invitrogen	A-21447
Donkey	Anti-guinea pig	Alexa Fluor 594	1:250	Jackson ImmunoResearch Laboratories	706-585-148
-	Streptavidin	Alexa Fluor 555	1:250	Molecular Probes	S32355
Goat	Anti-mouse IgG3	Alexa Fluor 488	1:250	Invitrogen	A21151
Goat	Anti-mouse IgG2a	Alexa Fluor 647	1:250	Invitrogen	A21241

Supplementary Table 3. qPCR Primers.

Gene Symbol	Full Name	Accession number	Forward primer (5'→3')	Reverse primer (5'→3')	Amplicon size (bp)
ACTB	Actin beta	XM_005340038.3	GCACTCTTCCAGCCTTCTT	CATAGAGGTCCTTGCGAATGT	106
OTX2	Orthodenticle homeobox 2	XM_005322736.3	AGGGTGCAGGTATGGTTTAAG	CGAGCTGGAGATGTCTTCTTT	116
RCVRN	Recoverin	XM_005332873.2	GCTCCTTCCAGATGATGAGAAC	GGTTCCCTCGATGAACTCTTG	112
OPN1SW	Opsin 1, short wave sensitive	XM_021722408.1	CCATTCCTGCCTTCTTCTCTAA	CCTACACACCATCTCCATGATAC	104
OPN1MW	Opsin 1, medium wave sensitive	NM_001282263.1	CAGTCGAGCATCTTCACCTATAC	GGTAATGTGGTACACCCATCTG	99
RHO	Rhodopsin	XM_005333784.3	CTTCACCTGGATCATGGCGT	GGGCGTGTAGTAGTCGATCC	109
CABP5	Calcium binding protein 5	XM_021734138.1	CTCATGAGGACAATGGGTTACA	CAGCTCCACGAAGTCATCAA	111
VSX1	Visual system homeobox 1	XM_005334613.2	CAGTGCTCAACTCCACAGAA	TCTTCACTTCCTGGTTTCCTTATC	106
RLBP1	Retinaldehyde binding protein 1	XM_005320129.2	TCAAGGCCATCCACTTCATC	CATGGACAAAGACCCTCTCAA	105
GFAP	Glial fibrillary acidic protein	XM_005328132.2	GAGTACCAGGACCTGCTTAATG	GTCTGCACTGGAATGGTGATA	107
